# The impact of human leukocyte antigen mismatching on graft survival and mortality in adult renal transplantation: A protocol for a systematic review and meta-analysis

**DOI:** 10.1097/MD.0000000000008899

**Published:** 2017-12-08

**Authors:** Xinmiao Shi, Wenke Han, Jie Ding

**Affiliations:** a Department of Pediatrics; b Institute of Urology, Peking University; c Department of Urology, Peking University First Hospital, Beijing, China.

**Keywords:** adult, graft survival, human leukocyte antigen, kidney transplant, meta-analysis, mismatching, mortality

## Abstract

**Background:**

Human leukocyte antigen (HLA) was important biological barrier to a successful transplantation. Quantitative evaluations of the effect of HLA mismatching on heart, liver, umbilical cord blood, and hematopoietic stem cell transplantation, have previously been reported. In new era of immunosuppression, the reported magnitude effect of HLA mismatching on survival outcomes of kidney transplantation was controversial. In addition, the current kidney allocation guideline recommendations in different countries were inconsistent in term of HLA mismatching. We undertake this study to conduct a systematic review and meta-analysis to assess the magnitude effect of HLA mismatching in adult kidney transplantation, with a particular focus on graft survival and mortality.

**Methods:**

The present systematic review and meta-analysis protocol was conducted following the Meta-analysis of Observational Studies in Epidemiology protocol (MOOSE-P) and the Preferred Reporting Items for Systematic Reviews and Meta-Analysis protocol (PRISMA-P). PubMed, EMBASE, Cochrane library Database will be searched without language restriction. Studies fulfill the following criteria will be eligible: included study cohorts comprising adult recipients; reported the association between HLA mismatching (per mismatches or HLA-A, -B, -DR mismatches) and posttransplant survival outcomes; provided effect estimates of hazard ratios (HRs) with 95% confidence interval (CIs). The incidence of measured outcomes was defined according to the European Renal Best Practice Transplantation Guidelines and Kidney Disease: Improving Global Outcomes Guidelines.

**Results:**

This study will quantitatively assess the association of HLA per mismatches, DR-antigen mismatches, A-antigen mismatches, and B-antigen mismatches with survival outcomes of overall graft failure, death-censored graft failure, all-cause mortality, and mortality with a functioning graft.

**Conclusion:**

This study will determine the issues on what extent HLA compatibility influenced recipient and graft survival and which HLA antigen plays a more important role in kidney transplantation.

**Systematic review registration:**

PROSPERO CRD42017071894.

## 1 Introduction

Renal transplantation is a more preferred option for end-stage renal disease (ESRD) than dialysis.^[1]^ In recent report of global database on donation and transplantation (www.transplant-observatory.org), around 80,000 renal transplants were performed annually.^[2]^ However, in 2016 United States Renal Data System (USRDS) Annual Data Report, the long-term survival benefit remained poor, with 10-year graft survival probabilities of 46.9% for cadaveric donor transplant.^[3]^

Human leukocyte antigen (HLA) was important biological barrier to a successful transplantation and has substantial impact on the prolongation of graft survival.^[4]^ The emergency of modern immunosuppressive agents minimized the effect of HLA compatibility. The US kidney allocation system was extensively modified to eliminated HLA-A similarity in 1995^[5]^ and HLA-B similarity in 2003.^[6]^ In the revised United Kingdom kidney allocation scheme, HLA-A matching is no longer considered.^[7]^ But several studies still demonstrated significant improvements in graft survival with a closely HLA-matched kidney. Recently survey from Massie et al^[8]^ with 106,019 recipients from Scientific Registry for Transplant Recipients (SRTR) data revealed that HLA-B and HLA-DR mismatches were associated with higher risks of all-cause graft failure. Australia and New Zealand Dialysis and Transplant Registry (ANZDTR) survey with 12,662 participants suggested that each incremental increase of HLA mismatches was significantly associated with a higher risk of graft failure and rejection.^[9]^ The latest European Renal Best Practice Transplantation Guidelines still recommended that matching of HLA-A, -B, and -DR whenever possible, while gave more weight to HLA-DR locus.^[10]^ So far, the issues on what extent HLA compatibility influenced patient and graft survival, and which HLA antigen plays a more important role, remains controversial. Here, we sought to conduct a systematic review and meta-analysis to quantitative assess the magnitude effect of HLA mismatching in adult kidney transplant recipients, with a particular focus on graft survival and mortality.

## 2 Methods

The study was registered in the PROSPERO international prospective register of systematic reviews (CRD42017071894). The protocol is performed in accordance with the meta-analysis of observational studies in epidemiology protocol (MOOSE-P)^[11]^ and the preferred reporting items for systematic reviews and meta-analysis protocol (PRISMA-P).^[12]^ Because this is a literature-based study, ethical approval is not required.

### 2.1 Literature search strategy and study selection

**Figure 1:**
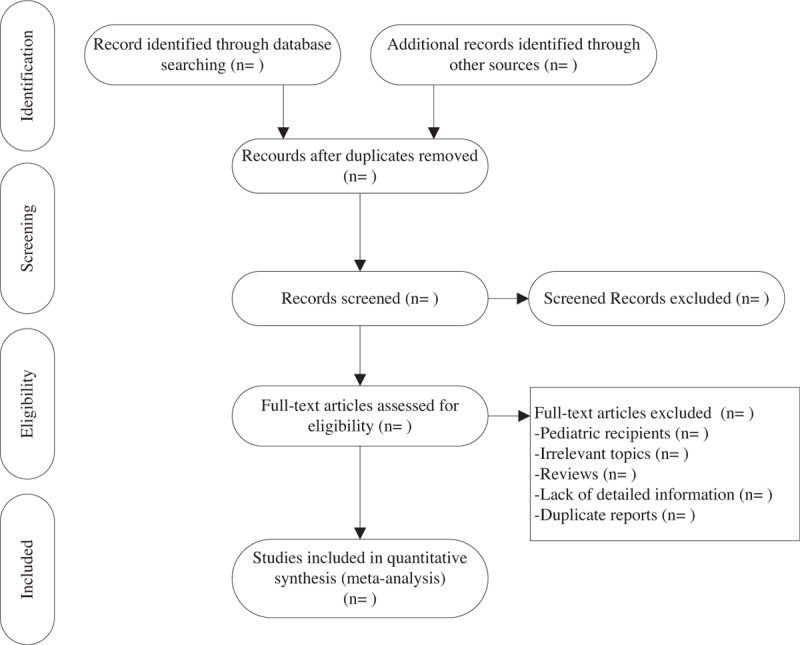
Predefined PRISMA flow chart. PRISMA = the preferred reporting items for systematic reviews and meta-analysis.

We will perform a comprehensive searched of PubMed, EMBASE, and the Cochrane Library, without language restriction. We used the following combinations of Medical Subject Heading (MeSH) terms and corresponding text-words: “kidney transplantation,” “renal transplantation,” “kidney transplant,” “renal transplant,” “human leukocyte antigen,” “HLA,” “mismatching,” “compatibility or incompatibility,” and all possible spellings of “graft survival” and “mortality” (Table 1). Reference lists of articles were manually screened to identify further relevant studies. The literature search was performed independently by 2 investigators (XS and XZ). The details of the selection process are shown in Fig. 1. Endnote X7 (Thomson Reuters, New York, NY) software was used to manage the studies that have been searched and remove duplicates. Differences were resolved by team discussion.

**Table 1:**
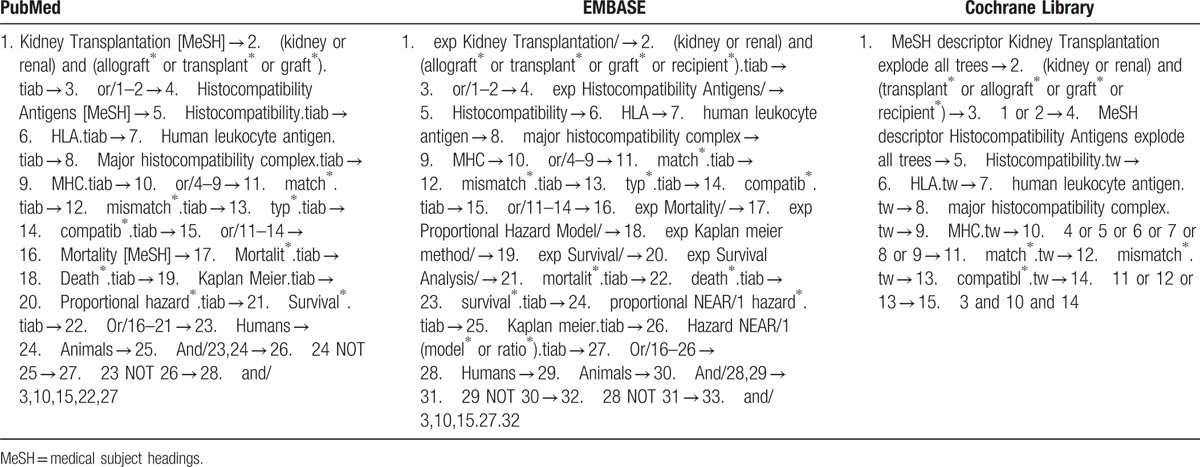
Search strategy for electronic database.

We included studies that included study cohorts that comprise adult recipients; reported associations between HLA mismatching and the posttransplant survival outcomes; and provided effect estimates of hazard ratios (HRs) with 95% confidence interval (CIs). We excluded publications reporting research on pediatric recipients or animals, in vitro research, studies on irrelevant topics, or studies lacking sufficient data (such as reviews, meta-analyses, case reports, case series, and technical descriptions). For studies covered overlapping data, we included the most recent and informative one. XS and XZ independently screened the titles and abstracts for eligibility. Discrepancies were resolved by team discussion.

### 2.2 Outcome measures

The priori primary clinical endpoint was overall graft failure; secondary clinical endpoints were death-censored graft failure, all-cause mortality, and mortality with functioning graft. The incidence of measured outcomes was defined according to the European Renal Best Practice Transplantation Guidelines and Kidney Disease: Improving Global Outcomes Guidelines.^[13,14]^

### 2.3 Data extraction

Data were recorded in a standardized Excel tables (Table 2), including the first author's name, publication date, study location, study design, cohort size, recipient age, sex distribution, duration, donor source, data source (multicentered or single-centered), follow-up, unadjusted and adjusted HRs of overall graft failure, death-censored graft failure and all-cause mortality per HLA-mismatch increased, and adjusted covariates in reported multivariable analysis. We contacted libraries abroad or corresponding author of relevant articles by email when detailed data for pooling analysis were unavailable.

**Table 2:**
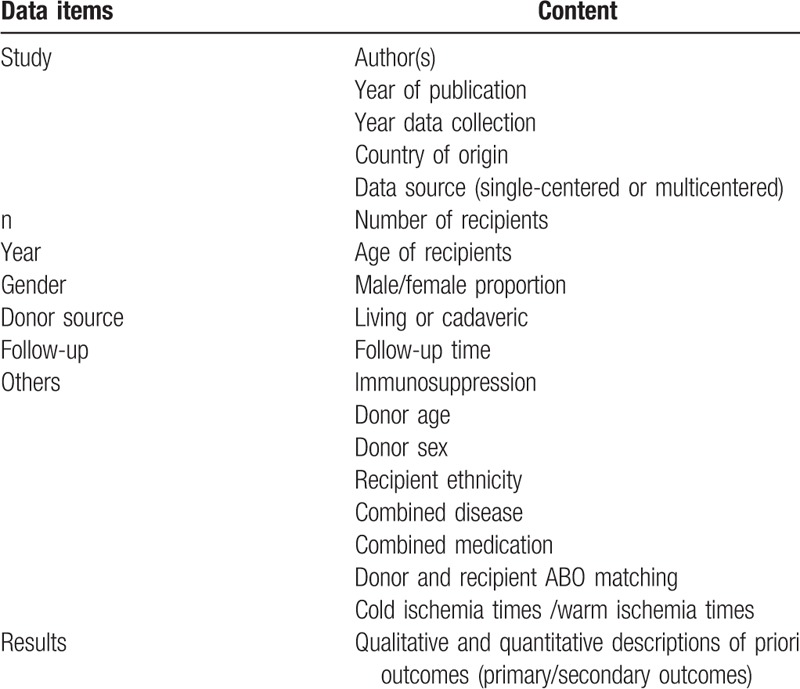
Data extraction variables.

### 2.4 Quality assessment

The methodological quality of included studies was described using the Newcastle-Ottawa Scale. High-quality studies were defined by a score of >5 points.^[15]^ Disagreements in the scores were resolved by team discussion.

### 2.5 Data synthesis

Hazard ratios (HRs) with corresponding 95% confidence intervals (CIs) were directly retrieved from each study. We chose HRs as the statistic estimates because they correctly reflect the nature of data and account for censoring. Cochran's *Q* test and *I*^2^-statistic were applied to assess heterogeneity between studies. The following criteria were used: *I*^2^ < 50%, low heterogeneity; 50–75%, moderate heterogeneity, and >75%, high heterogeneity.^[16,17]^ When significant heterogeneity was found between studies (*P* < .10 or *I*^2^ > 50%), the effect estimates were calculated using a random-effects model and the DerSimonian–Laird method;^[18]^ otherwise, a fixed-effects model with the Mantel–Haenszel method was used.^[19]^ Subgroup analyses included recipient sample size, the nature of data (univariable-unadjusted vs multivariable-adjusted effect estimates), donor source (cadaveric, living, and living + cadaveric), data source (multicentered vs single-centered), and ethnicity. A sensitivity analyses were performed by omitting one study at a time and then reanalyzing the data to assess the change in effect estimates. To further explore heterogeneity, a random-effects univariate meta-regression was conducted when at least 10 studies were available. For outcomes of at least 10 studies included, publication bias was assessed by funnel plot and Egger test.^[20]^ Egger test with 2-tailed significance level of 0.10 was considered to be statistically significant. Analyses were performed using STATA software, version 13.0 (STATA Corporation, College Station, TX).

## 3 Discussion

To our knowledge, this is the first protocol of systematic review and meta-analysis to assess the effect of HLA mismatching on posttransplant survival outcomes in the adult kidney transplantation, providing a detailed summary of the available evidence.

Human HLA genes, located on chromosome 6, code for 3 major class I alleles (HLA-A, -B, -C), and 3 major class II alleles (HLA-DR, -DQ, -DP). Polymorphisms in HLA, especially HLA-A, -B, and -DR loci, are important biological barriers to a successful transplantation.^[3,21]^ As closely HLA-matched graft is less likely to be recognized and rejected, HLA mismatching has a substantial impact on prolongation of graft survival.^[22]^ Quantitative assessments of the effect of HLA mismatching on heart, liver, umbilical cord blood, and hematopoietic stem cell transplantation, have already been reported. But a quantitative analysis for the associations of HLA compatibility and posttransplant survival outcomes in adult renal transplantation, the most common organ transplant with the largest subjects of recipients, is still lacking.

With the emergence of potent immunosuppressive agents that steadily improved graft survival rates, the impact of HLA compatibility seems to be minimized.^[3,23]^ Different regions or countries (European, US, UK, Australia, Israel, etc.) reported different kidney allocation guideline recommendations based on HLA-compatibility.^[6–8,24,25]^ But the recommendations were different. Now, it was necessary to conduct comprehensive quantitative analyses to explore the magnitude effect of HLA compatibility on graft and recipients survival outcomes in kidney transplantation.

The strengths of our meta-analysis are strict study design and using hazard ratios (HRs) as statistic estimates to more correctly reflect the nature of data and account for censoring. However, the absence of randomized controlled trials was a limitation of our study. The findings of this systematic review could be of interest for nephrologist, kidney transplant surgeon, and kidney allocation policy-makers, providing evidence as a basis for more judicial kidney allocation to achieve the goal to make the kidney last as long as possible.

## Acknowledgments

The authors thank the National Key Research and Development Program of China (2016YFC0901505) and the registry of rare diseases in children, Beijing key laboratory of molecular diagnosis and study on pediatric genetic diseases (BZ0317) for the support.
